# Overcoming Operational Challenges to Ebola Case Investigation in Sierra Leone

**DOI:** 10.9745/GHSP-D-17-00126

**Published:** 2017-09-27

**Authors:** Samuel T Boland, Erin Polich, Allison Connolly, Adam Hoar, Tom Sesay, Anh-Minh A Tran

**Affiliations:** a London School of Hygiene & Tropical Medicine, London, United Kingdom.; b GOAL Global, Dublin, Ireland.; c Ministry of Health and Sanitation, Freetown, Sierra Leone.

## Abstract

The Ebola virus disease (EVD) epidemic that hit West Africa in 2013 was the worst outbreak of EVD in recorded history. While much has been published regarding the international and national-level EVD responses, there is a dearth of literature on district-level coordination and operational structures, successes, and failures. This article seeks to understand how the EVD response unfolded at the district level, namely the challenges to operationalizing EVD surveillance over the course of the outbreak in Port Loko and Kambia districts of Sierra Leone. We present here GOAL Global's understanding of the fundamental challenges to case investigation operations during the EVD response, including environmental and infrastructural, sociocultural, and political and organizational challenges, with insight complemented by a survey of 42 case investigators. Major challenges included deficiencies in transportation and communication resources, low morale and fatigue among case investigators, mismanagement of data, mistrust among communities, and leadership challenges. Without addressing these operational challenges, technical surveillance solutions are difficult to implement and hold limited relevance, due to the poor quality and quantity of data being collected. The low prioritization of operational needs came at a high cost. To mediate this, GOAL addressed these operational challenges by acquiring critical transportation and communication resources to facilitate case investigation, including vehicles, boats, fuel, drivers, phones, and closed user groups; addressing fatigue and low morale by hiring more case investigators, making timely payments, arranging for time off, and providing meals and personal protective equipment; improving data tracking efforts through standard operating procedures, training, and mentorship to build higher-quality case histories and make it easier to access information; strengthening trust in communities by ensuring familiarity and consistency of case investigators; and improving operational leadership challenges through meetings and regular coordination, establishing an active surveillance strategy in Port Loko, and conducting an after-action review. Resolving or addressing these challenges was of primary importance, and requisite for the implementation of technical epidemiological complements to EVD case investigation.

## Abstract (precis)

Deficiencies in transportation and communication, low frontline staff morale, and mistrust among communities, among other operational challenges, greatly limited Ebola case investigation in Sierra Leone. Recommendations for future outbreaks: (1) timely compensation for frontline staff, (2) context-appropriate transportation and communication resources, (3) systematic data collection, storage, and retrieval systems, (4) sound linkages between frontline staff and communities, (5) daily meetings between frontline staff and epidemiologists, (6) clear and appropriate operational chain of command, and (7) political and funding support to operational agencies.

## INTRODUCTION

The West African Ebola virus disease (EVD) epidemic began in Meliandou, Guinea, in December 2013, before spreading to Liberia and Sierra Leone in March and May 2014, respectively. By July 2014, EVD had spread beyond containment, and on August 8, 2014, the World Health Organization (WHO) declared a “Public Health Emergency of International Concern.”^1^

While there is new literature on the scale of the outbreak, international-level failures, and EVD clinical features and transmission chains to help inform and contextualize these numbers, there is a dearth of literature on the operational details of the EVD response at the district level in Sierra Leone.^2^ These details include day-to-day activities and the difficulties faced when mounting a response to a large-scale outbreak in resource-limited settings. This article attempts to help fill this gap, drawing on the authors' collective 64 months of experience with the EVD response efforts in Sierra Leone. Specifically, we discuss case investigation operations in Port Loko and Kambia districts of Sierra Leone.

Emergency bylaws enacted in August 2014 required the investigation of all deaths and cases of sickness, among other exceptional legal demands.^3^ Fulfilling this requirement and effectively managing the EVD outbreak required a well-trained and capacitated disease surveillance structure to conduct the following components:

- **Case investigation:** Conduct in-person assessments of all persons reported ill or deceased to determine if they met the case definition for suspected EVD, which is decided based on the confluence of 15 possible symptoms and history of EVD contact. Refer suspected cases to EVD treatment centers. Document all known contacts of the suspected EVD case and search for all missing contacts.
- **Dead body swabbing:** Using oral swab specimens, test all dead bodies for post-mortem EVD before burial. If specimen tests EVD-positive, conduct follow-up investigation and contact tracing.
- **Contact tracing:** Monitor anyone who has direct contact with an individual with EVD for signs and symptoms of the virus during the 21-day incubation period. Note that the term “contact tracing” in the Sierra Leonean context refers to the monitoring of known contacts, rather than elicitation and location of previously unknown contacts.

Emergency bylaws enacted in August 2014 required the reporting and investigation of all deaths and cases of sickness, among other exceptional legal demands.

EVD surveillance requires all 3 of these complementary and co-dependent components; however, this manuscript focuses on the first component—case investigation and specifically its operational needs.

In October 2014, the National Ebola Response Centre (NERC) was created to provide a central, coordinated response to EVD at the national level.^4^ Corresponding District Ebola Response Centres (DERCs), responsible for local EVD response operations, rolled out to the country's 13 districts shortly thereafter.^5^ DERCs were staffed by the Republic of Sierra Leone Armed Forces, the British Armed Forces, the UK Department for International Development (DFID) and the UK Stabilisation Unit, the U.S. Centers for Disease Control and Prevention (CDC), the respective district medical officer (DMO) and members of their district health management team (DHMT), NGOs, and several United Nations agencies, including the Mission for Ebola Emergency Response and WHO.

However, even with this structure in place, conducting case investigation, dead body swabbing, and contact tracing was an enormous effort considering the scale of the outbreak. Sierra Leone has high levels of all-cause morbidity and mortality rates,^6^ and EVD presents many clinical similarities to endemic diseases like Lassa fever and malaria, the latter alone accounting for 50% of health facility outpatient visits.^7^ Indeed, by late 2014, Port Loko district experienced one of the highest EVD caseloads in the country, with nearly 100 laboratory-confirmed cases per week.^8^

Even with a robust national structure in place, conducting Ebola disease surveillance was an enormous effort considering the scale of the outbreak.

The high burden of cases, requiring daily investigation, demanded substantial logistical resources and an operational framework—both of which were lacking in the earliest stages of the outbreak—and DERCs remained unable to contain the rapid spread of EVD. Delays in scaling up an international public health emergency response—for which there was little precedence—and a heavy focus on providing technical epidemiological support rather than operational and logistical support, exacerbated these challenges.

Rapid field epidemiology training by WHO and the CDC in late 2014 developed a much-needed cadre of Sierra Leonean case investigators (also referred to in-country as district surveillance officers), complemented by a cohort of expatriate WHO and CDC epidemiologists. However, operational support to facilitate their work during the height of the outbreak was still insufficient. EVD case investigation quality and efficiency thus remained insufficient through 2014. In Port Loko district during the month of December 2014, 22% of confirmed EVD cases were found dead at the time of investigation, only 26% of confirmed EVD cases had a known source case, and the percentage of confirmed EVD cases previously identified as EVD contacts was a mere 15%.^9^

**Figure:**
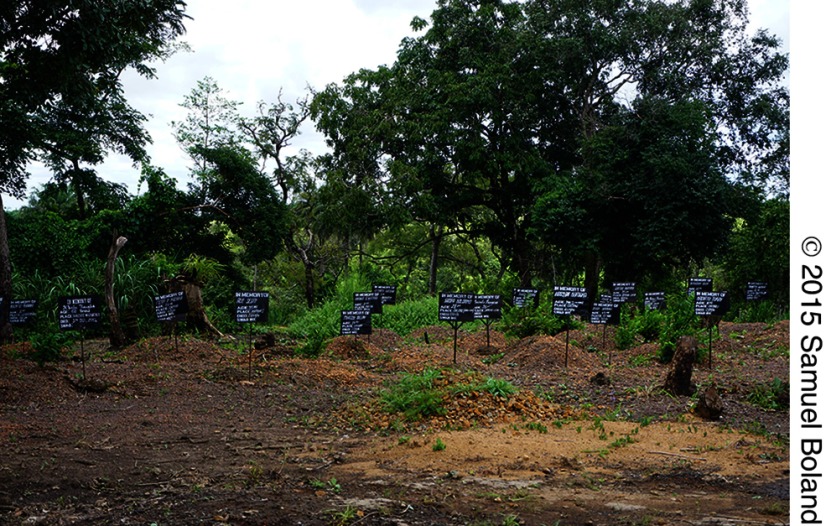
A safe burial cemetery in Kambia, Sierra Leone.

## METHODS

In December 2014, the Port Loko DERC requested NGO support for EVD surveillance from the Ebola Response Consortium, an NGO consortium in Sierra Leone led by the International Rescue Committee. GOAL Global thus began supporting EVD surveillance in Port Loko in January 2015, with DFID funding. The same request was made in Kambia in March 2015, with GOAL support beginning in April 2015. What follows are the challenges encountered in conducting EVD case investigation in Port Loko and Kambia, and the work of GOAL in collaboration with various DERC and DHMT stakeholders to address and effectively operationalize EVD surveillance activities in these 2 districts. A summary of challenges and interventions is presented in the Table.

**TABLE.** Challenges and Solutions to Conducting Ebola Virus Disease Case Investigation, Sierra Leone

Type	Challenges	Solutions
**Environmental and Infrastructural**		
Transportation	Lack of vehicles, poorly maintained low-quality vehicles, and lack of fuel posed challenges for case investigators to cover daily alerts of sickness and death in the 2 districts. Poor-quality unpaved roads, riverine areas, and the rainy season from May to September further complicated response efforts.	GOAL collaborated with DFID, NERC, WHO, and other NGOs to provide high-quality off-road vehicles, along with fuel, drivers, and other logistical support. GOAL rented 12 vehicles in Port Loko and Kambia, and advocated with WHO and NERC to provide additional vehicles. GOAL requested authorization and funding from DFID for Catholic Relief Services in Port Loko and GOAL in Kambia to fuel the additional vehicles. GOAL advocated and helped facilitate the acquisition of boats from the Republic of Sierra Leone Armed Forces to facilitate access to riverine areas in Kambia.
Communication	Deficiencies in telecommunications infrastructure made it difficult for communities to raise sickness and death alerts, particularly in rural areas. Case investigators could not reliably locate alerts and often failed to communicate important information due to unspecific reported locations, lack of phone credit, or unreliable network coverage.	GOAL distributed cellular phones, phone credit, and satellite phones to case investigators and their coordinators in the DHMT and DERC. GOAL provided closed user groups to all case investigation teams and selected individuals in the DERC, enabling free unlimited calling between case investigators, their supervisors, and epidemiology teams in WHO and CDC.
Data quality and management	Mismanagement of investigation materials posed a challenge to locating specific information, tracking efforts, and building case histories. Discrepancies occurred at the field and district levels, and no formal filing mechanism existed for completed case investigation forms. Inconsistency in naming conventions, spelling, and characterization of residence made matching documents difficult.	GOAL developed standard operating procedures in collaboration with all surveillance stakeholders. Standard operating procedures ensured that case investigation forms were collected, stored, and organized in a retrievable manner. GOAL hired data managers in both the Port Loko and Kambia DERC to file and immediately digitize this information in real time. WHO established an after-action review in Port Loko in collaboration with GOAL, CDC, and the DHMT to review case investigation information and data at the end of each day.
Personal safety and fatigue	Case investigators were unable to eat during their long work day due to dangers of purchasing food from high-risk communities, stigma from communities who feared them, a lack of personal funds, and insufficient time.	GOAL immediately provided daily take-away breakfast and lunch to all case investigators. Case investigators received hand sanitizer and personal protective equipment to help prevent EVD infection, and rain gear allowed for easier movement of personnel during the rainy season.
**Sociocultural**		
Community trust	A lack of community trust in response staff and enormous stigma resulted in difficulties conducting case investigations, lack of truthful information, and sometimes violence. Case investigators rarely returned to the same communities each day and generally did not work in their own communities.	GOAL assigned a dedicated team for each of Port Loko's and Kambia's chiefdoms to ensure familiarity and consistency. To the extent possible, case investigators were assigned to work in their chiefdom of origin.
Traditional healers	A lack of trust in facility-based health care and fear of nosocomial infections drove many to seek health care from traditional healers. Despite the fact that traditional healers were legally banned from practicing and required to report cases of illness, many people disregarded the bylaws despite fears of punitive measures. Cases of sickness and death went unreported, and traditional healers fueled new EVD clusters when they contracted the disease from their patients.	DERC stakeholders, including GOAL, attempted to formally involve traditional healers as public health agents in the EVD response. However, due to the illegality of their work under the national bylaws, there was strong political hesitation to permit activity that appeared to legitimize the trade. As such, further efforts to include traditional healers were not pursued.
**Political and Organizational**		
Management structures	In Port Loko, GOAL became the lead operational agency and coordinator of the surveillance pillar. However, in Kambia, WHO continued leading surveillance, directing operational activities, and overseeing logistical needs despite not controlling operational resources. Crucial operational adjustments in Port Loko were therefore not easily implemented in Kambia. No single organization was identified as the lead agency for case investigators, and therefore no single point of advocacy existed to resolve their needs, complicating the resolution of problems.	Leadership challenges in Kambia were addressed to some degree through relationship building and regular coordination with technical leads and DERC management. GOAL, WHO, and CDC developed an active surveillance strategy in Port Loko to address the high proportion of EVD cases identified post-mortem or with no known source case.
Human resources	The lack of sufficient case investigators and the work fatigue that resulted were among the biggest challenges facing case investigators. Funding constraints and a perceived lack of need at the NERC resulted in a national directive that prevented hiring new surveillance staff.	GOAL advocated with DFID and NERC to bring in additional human resources. The DHMT, GOAL, WHO, and CDC trained the new case investigators. Mentorship in the field reinforced the training. A rotation system was implemented to provide time off to address surveillance efficiency, quality, and case investigator work fatigue.
Compensation	Case investigators averaged more than a month of missed pay per person and could sometimes not afford to buy food or pay rent. At the national level, case investigators were sometimes incorrectly relegated to lower pay categories and clerical errors resulted in their removal from payroll. Threats of strikes were frequent and morale was extremely low.	Resolving case investigator salary issues was a protracted and complicated process. Initially, NERC paid all case investigators, with funding from the World Bank. Ultimately, GOAL secured DFID funding and NERC permission to pay all case investigators directly in both districts beginning in July 2015.
Inter-pillar coordination	Many of the 11 vertical pillars of operation at DERCs performed complementary work. Horizontal integration and cooperation between pillars was profoundly challenging, which often resulted in a lack of effective cooperation between them.	Meetings were established to create horizontal linkages between the pillars in Port Loko in January 2015 and in Kambia in April 2015. GOAL attempted to reinforce horizontal communication by developing an EVD response framework, which was not fully realized because it was developed late in the response.

Abbreviations: CDC, U.S. Centers for Disease Control and Prevention; DERC, District Ebola Response Centre; DFID, UK Department for International Development; DHMT, district health management team; EVD, Ebola virus disease; NERC, National Ebola Response Centre; WHO, World Health Organization.

GOAL Global began supporting EVD surveillance in two districts—Port Loko and Kambia—in January 2015 and March 2015, respectively.

This insight draws from our experiences working in the EVD response, as well as a survey of 42 Port Loko and Kambia case investigators conducted by 2 of the authors in December 2015. The University of Chicago Social and Behavioral Sciences Institutional Review Board reviewed and approved the survey.

We acknowledge the following limitations of this analysis: All data presented here were official data, or data collected by the authors themselves during the response, with all efforts made to portray an accurate picture. However, data quality is admittedly limited due to the crisis conditions of the EVD response. Additionally, this article represents the viewpoints of a limited number of professionals working in Port Loko and Kambia districts of Sierra Leone during this time—only several among thousands of national staff and many tens of international responders who worked in these districts during the outbreak.

**Figure:**
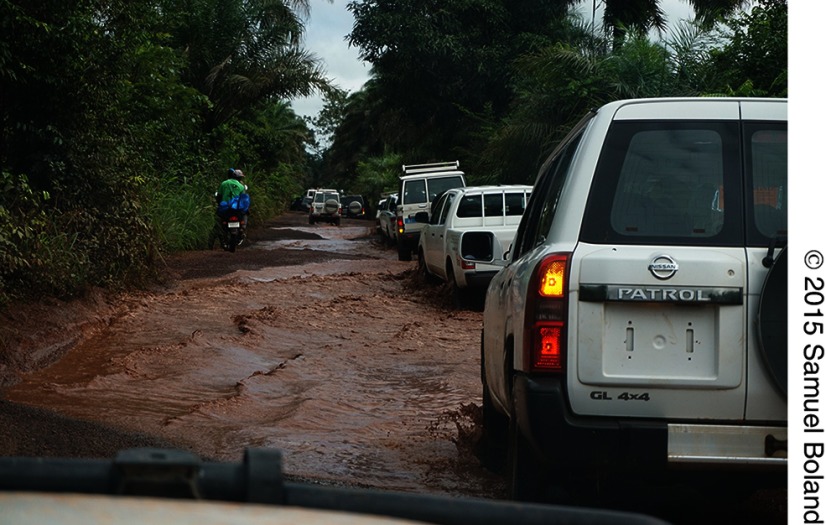
In Sierra Leone, case investigators had to navigate unpaved roads and riverine areas.

## CHALLENGES TO CONDUCTING EVD CASE INVESTIGATION

### Environmental and Infrastructural Challenges

Case investigator transportation presented a substantial challenge to EVD surveillance. In Port Loko and Kambia, only 7 and 3 vehicles, respectively, were available for case investigation as of December 2015 to cover an average of 43.3 daily alerts of sickness and death in Port Loko and 8.1 in Kambia,^10^ among a total population of 900,000 people over a total area of 8,827 square kilometers.^11^^,^^12^ This created major logistical challenges to response capacity.

In addition to the challenge presented by lack of vehicles, both Port Loko and Kambia have poor-quality unpaved roads and a complicated latticework of riverine areas. Accessibility difficulties are exacerbated during the rainy season from May to September, when the 2 districts each receive between 2 and 4 meters of rain.^13^ Poorly maintained and poor-quality vehicles were inadequate for such harsh conditions and prevented timely and efficient case investigation when they broke down or could not navigate roads. A lack of reliable fuel supply further hampered case investigation efforts.

Severe communication challenges also impeded case investigation. Deficiencies in Sierra Leone's telecommunications infrastructure meant it was often difficult for communities to raise sickness and death alerts, particularly in rural areas. As such, the flow of information necessary for EVD surveillance was often limited at its point of origin.

Severe communication challenges limited the flow of information necessary for EVD surveillance.

However, if communities raised an alert, there was no guarantee that a case investigator would successfully locate the alert, as the reported locations were often unspecific and investigators often lacked phone credit or found themselves out of network coverage to call for clarification. Additionally, case investigators often failed to communicate important information back to the DERC because of these same reasons. The delay in both accessing cases and reporting on them due to telecommunications challenges resulted in protracted and haphazard case investigations and follow-up.

Data management of investigation materials from the field and within the DERC also posed a challenge to effectively tracking and building case histories, with discrepancies occurring both in the field and at the DERC. No formal filing mechanism existed for completed case investigation forms, which were in hard copy only and were often scattered across multiple DERC offices. Locating specific information was therefore challenging and time-consuming; this was further complicated by Sierra Leonean naming conventions, variations in spelling, and characterization of residence, which made matching documents exceptionally difficult. The need for a systemized retrieval mechanism was crucial because an EVD case was generally not laboratory-confirmed on the day it was investigated, when the case investigation form was completed. As such, if a positive case result returned from the laboratory, locating the initial case investigation form from a previous day often proved difficult. A case investigation form duplicated from memory usually resulted in a lower-quality case report.

No formal filing mechanism existed for completed case investigation forms and locating information was challenging and time-consuming.

Lastly, case investigators were unable to eat during their long work day due to dangers of purchasing food from high-risk communities, stigma from communities who feared EVD responders, a lack of personal funds to do so, and insufficient time. Case investigators complained of fatigue due to insufficient nutrition, which resulted in limiting work efficiency and rigor.

### Sociocultural Challenges

Sociocultural challenges further limited EVD surveillance efficacy. Enormous stigma against EVD response workers and a powerful lack of trust in EVD response staff resulted in difficulties generating high-quality case investigations and sometimes resulted in violence against case investigators.^14^ Due to insufficient human resources and logistical capacity, case investigators rarely returned to the same communities each day, but instead responded to alerts ad hoc. Nor was it standard procedure for case investigators to operate within their own community. Thus, communities often regarded them with suspicion as outsiders. For instance, in April 2015, community members chased case investigators out of a remote village in Kambia and threatened them with machetes when they were dispatched to investigate an alert.

Case investigators rarely returned to the same communities or operated within their own communities, which resulted in lack of trust and sometimes violence.

**FIGURE 1:**
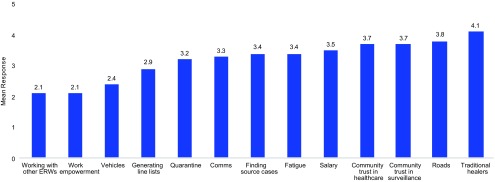
Challenges Identified by Case Investigators as Most Significant^a^ to EVD Case Investigation, Port Loko and Kambia Districts, Sierra Leone (N=42) Abbreviations: ERW, Ebola response workers; EVD, Ebola virus disease. ^a^ On a scale of 1 to 5 (1=not significant at all, 5=most significant).

When case investigators in Port Loko and Kambia were asked to rank the challenges they considered most significant to their work, 77% included “community trust in surveillance” as 1 of their top 3 challenges (Figure 1). This lack of trust led to lower-quality investigations, as communities were reticent to provide truthful information to case investigators.

Additionally, a lack of trust in facility-based health care and fear of nosocomial infections drove many to seek health care from traditional healers. While traditional healers were legally banned from practicing under the emergency bylaws and required to report any cases of illness,^3^ many disregarded the bylaws and continued treating patients without integration with EVD surveillance or response activities. As such, many instances of sickness and death remained unreported, and the high-risk population of traditional healers fueled new EVD clusters when they contracted EVD from their patients. Traditional healers and those with connections to them often were not transparent with case investigators for fear of punitive measures for violating the emergency bylaws.

### Political and Organizational Challenges

Some of the greatest challenges that faced EVD surveillance in Port Loko and Kambia were political and organizational challenges. WHO district offices were intended to provide technical support and offer an advisory role to DERC operations. In the early days of the response and in the absence of widespread external support, WHO also provided operational and logistical support for many DERC activities. This engendered a reliance within the DERC on WHO as both an advisory and operational body, despite the WHO Assistant Director-General Dr. Bruce Aylward attesting that the “organization … was not designed to be an operational field-based organization … play[ing] such a role.”^15^

When GOAL began supporting Port Loko case investigation in January 2015, it was quickly identified as the lead operational agency and coordinated the surveillance pillar (1 of 11 organizational pillars of operation within DERCs), collaborating closely with WHO and CDC as technical leads. In Kambia, due to a different response trajectory and political climate, WHO maintained coordination leadership of the surveillance pillar, directed operational activities, and oversaw logistical needs despite not controlling operational resources. The difference in these 2 management structures meant that operational adjustments in Port Loko identified as crucial and effective (see Interventions) were not easily implemented in Kambia, a political challenge that frequently limited response efficacy and efficiency.

The difference in operational management structures between the 2 districts meant that crucial operational adjustments in Port Loko were not easily implemented in Kambia.

This management structure of the surveillance pillar in Kambia contributed to challenges with coordination. No single organization was identified as the lead agency for case investigators, and therefore no single point of advocacy existed to resolve their needs, limiting the resolution of various identified and serious problems. In contrast, these concerns were addressed more efficiently in Port Loko, where management and organizational responsibilities were more clearly delineated.

Human resources, particularly the lack of sufficient case investigators and the work fatigue that resulted, were among the biggest challenges facing EVD case investigation in both Port Loko and Kambia. Throughout much of the outbreak, case investigators worked 7 days a week, without scheduled time off or periods of rest. From an operational standpoint, this was necessary as human resources were badly lacking. However, 21% of case investigators surveyed considered work fatigue as 1 of the 3 biggest challenges to their work (Figure 1). Funding constraints and a perceived lack of need at the NERC resulted in a national directive that prevented the hiring of new surveillance staff after March 2015. This became problematic, particularly in Kambia when GOAL and the DERC leadership identified an immediate need to increase the number of investigators from 3 to 9 in April 2015 to effectively address the persistent EVD caseload in Kambia and the high risk of EVD importation from neighboring Guinea.^16^

The lack of sufficient case investigators and the work fatigue that resulted were among the biggest challenges facing EVD case investigation in both Port Loko and Kambia.

Timely compensation was considered to be another major challenge by case investigators: 53% of case investigators included compensation as 1 of the 3 biggest challenges to their work (Figure 1). When asked “What are some things NERC could have done better?” 88% of case investigators listed late or non-compensation.

A letter dated February 16, 2015, from the Port Loko DMO to NERC listed a backlog of 92.4 million SLL (approximately US$18,500) among the district's 53 case investigators, averaging more than 1 month of missed pay per person, dating back to October 2014 (Alfred Kamara, written communication, February 16, 2015). These payments were not distributed until May 2015. In a low-income country where personal savings are nominal, case investigators sometimes complained that they could not afford to buy food or pay rent due to lack of payment. Additional issues at the national level included erroneous relegation of case investigators to lower pay categories and clerical errors that removed personnel from payroll. Threats of strikes were frequent, and morale was extremely low.

Late payments, non-compensation, and clerical errors related to pay resulted in threats of strike and extremely low morale among case investigators.

Lastly, the organizational design of the DERC itself challenged collaborative work. DERCs were organized by 11 vertical pillars of operation, each with its own function and management. Many pillars performed complementary work; however, horizontal integration and cooperation was profoundly challenging, as doing so could appear to encroach on identified management responsibility. This often resulted in a lack of effective cooperation between pillars.

## INTERVENTIONS TO ADDRESS OPERATIONAL EVD CASE INVESTIGATION CHALLENGES

Without addressing operational challenges, technical surveillance solutions are difficult to implement and hold limited relevance, due to the poor quality and quantity of data being collected to direct more technical and strategic solutions. In the early months of the Port Loko and Kambia DERC-led responses, epidemiological analysis was prioritized over operational needs. This lower prioritization came at a high opportunity cost, leading to a decrease in operational capacity and efficacy of interventions intended to resolve challenges detrimental to case investigation. To mediate this, GOAL addressed these operational challenges as outlined below, corresponding to the aforementioned categories of challenges.

### Environmental and Infrastructural Interventions

First, an intervention was necessary to provide case investigators with the basic resources they required to continue working. GOAL immediately provided daily take-away breakfast and lunch to all case investigators to prevent hunger, fatigue, and EVD exposure from purchasing food in high-risk communities and to avoid stigma faced in communities during investigations. Case investigators received hand sanitizer and appropriate personal protective equipment to help prevent EVD infection, and rain gear allowed for easier movement of personnel in flooded areas. Case investigator morale improved rapidly as a result.

To address transportation issues, GOAL collaborated with DFID, NERC, WHO, and other NGOs to provide high-quality off-road vehicles to case investigation teams in both Port Loko and Kambia, along with the requisite fuel, drivers, and other logistical support. GOAL rented 12 vehicles in Port Loko and Kambia, and advocated with WHO and NERC to provide additional vehicles for case investigation. GOAL asked DFID for authorization and funding for Catholic Relief Services as the district fleet management agency in Port Loko and GOAL in Kambia to source and provide fuel for the additional vehicles. This allowed the assignment of at least 1 case investigation team to each of the 2 district's chiefdoms. Boats were needed to facilitate access to riverine areas, which were successfully acquired from the Republic of Sierra Leone Armed Forces. These measures improved the timeliness, quality, and efficiency of case investigation activities in areas that were previously difficult or impossible to access.

GOAL collaborated with other agencies to provide high-quality off-road vehicles, fuel, drivers, and logistical support, as well as boats for riverine areas.

GOAL distributed cellular phones, phone credit, and satellite phones, where necessary, to case investigators and their coordinators in the DHMT and DERC, to ensure the timely communication of information between the DERC and the field. Closed user groups were provided to all case investigation teams and selected individuals in the DERC, enabling free unlimited calling between case investigators, their supervisors, and epidemiology teams in WHO and CDC. These measures also facilitated more timely requests, and ultimately, responsiveness from case investigators to the DERC for resources such as the dispatch of ambulance or burial teams.

Case investigators and coordinators in the DHMT and DERC received phones, phone credit, satellite phones, and closed user groups to ensure timely communication.

WHO established an after-action review in Port Loko in collaboration with GOAL, CDC, and the DHMT in January 2015, to review case investigation information and data at the end of each day. The after-action review provided an opportunity for ongoing training as epidemiologists reviewed cases with case investigators and provided guidance on how to improve subsequent investigations. The after-action review also ensured all surveillance information and case investigation forms from the field were systematically returned to the DERC each day, as well as provided a daily forum to raise operational concerns. This effective measure was immediately implemented by GOAL and other surveillance pillar partners in Kambia when their support began in April 2015.

**FIGURE 2:**
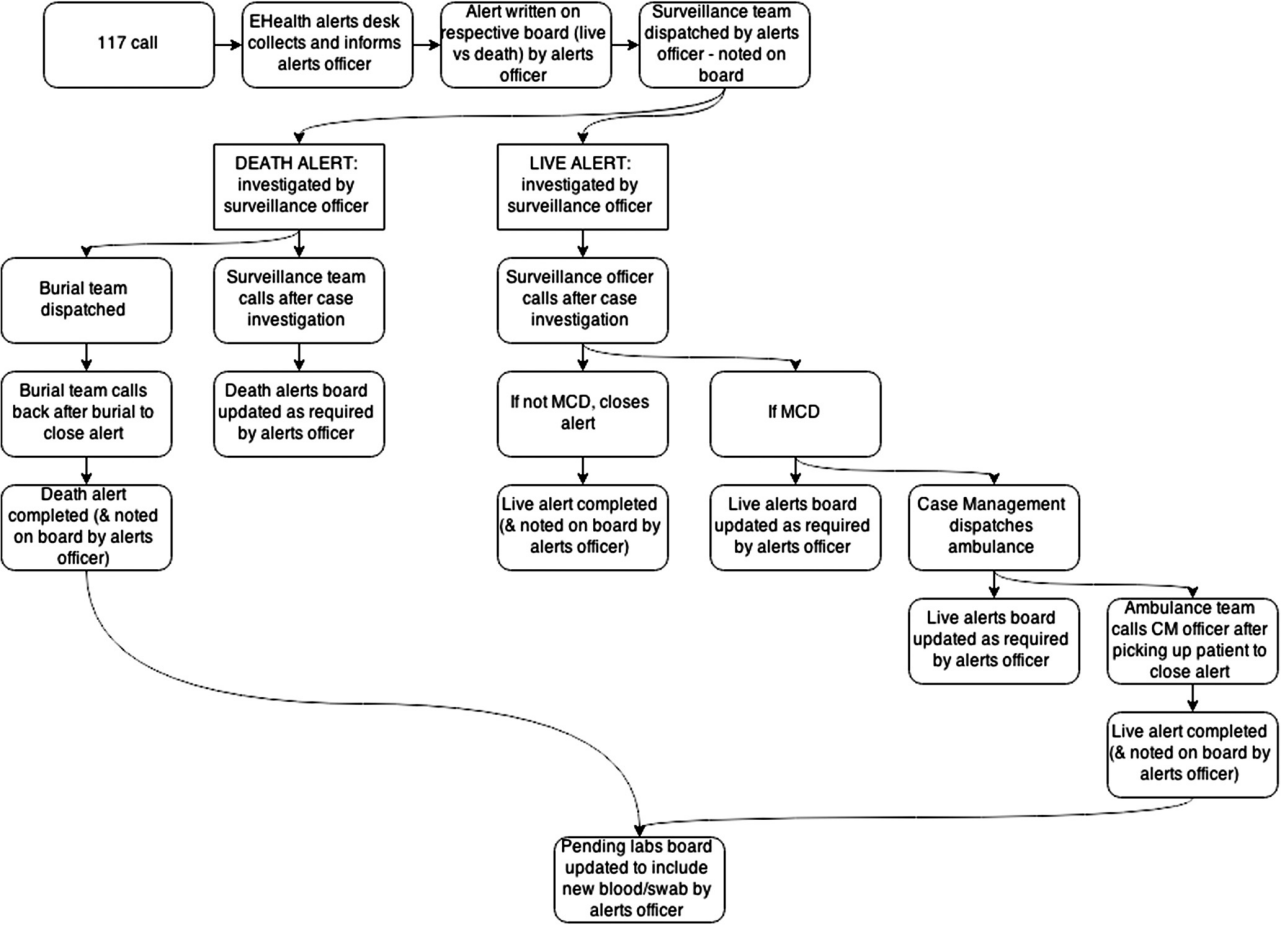
Flow Chart of Ebola Disease Alert Response, Sierra Leone Abbreviations: CM, case management; EVD, Ebola virus disease; MCD, meets case definition. Notes: A “117 call” is the national EVD alert hotline, the mechanism by which most occurrences of sickness and death were reported to the District Ebola Response Centres (DERCs). “EHealth” is an NGO that supported the DERC. This flow chart is part of the standard operating procedures developed by Samuel Boland on behalf of GOAL.

Challenges surrounding data quality and coordination were also addressed by developing various standard operating procedures in collaboration with all surveillance stakeholders, including the basic structure of alert response by case investigators (Figure 2). Standard operating procedures were also put in place to ensure case investigation forms were collected, stored, and organized in a retrievable manner. All information was consolidated in an organized filing cabinet. GOAL hired data managers in both the Port Loko and Kambia DERCs to file and immediately digitize this information in real time. This allowed for timely retrieval and communication of surveillance data to relevant stakeholders and EVD response workers, including case investigators.

### Sociocultural Interventions

Assigning 1 dedicated team for each of Port Loko's and Kambia's chiefdoms enabled familiarity and consistency between communities and the case investigators working within them. To the extent possible, case investigators were assigned to work in their chiefdom of origin. As a result, community trust in case investigators improved in both districts, and case investigators could thus be in regular conversation with local leaders such as paramount and village chiefs, teachers, health facility staff, and traditional healers. Additionally, data quality increased as case investigators became experts in specific EVD cases and transmission chains. This occurred because of the geographic consistency of case investigator deployment and the increased understanding of local cultural contexts surrounding a particular transmission chain.

Case investigators worked in dedicated chiefdoms to ensure familiarity and consistency, which strengthened trust among communities.

Several DERC stakeholders, including GOAL in Port Loko in June 2015, attempted to formally involve traditional healers as public health agents in the EVD response. However, despite the potential efficacy of their inclusion, due to the illegality of traditional healers' work under the national bylaws, there was strong political hesitation to permit activity that appeared to legitimize the trade.^17^ As such, further efforts to include traditional healers in the EVD response were not pursued.

### Political and Organizational Interventions

Additional human resources were brought in to address the issue of work fatigue in both Port Loko and Kambia. GOAL advocated for these additional case investigators with DFID and NERC, who agreed to increase case investigators in both districts. In July 2015, case investigators increased from 25 to 65 in Port Loko, and from 3 to 9 in April 2015 in Kambia— with a further increase from 9 to 21 in July 2015.^11^^,^^12^ The number of case investigators is relative to the size of the districts—Port Loko has an estimated population of 550,000 and an area of 2,208 square miles split among eleven chiefdoms, whereas Kambia has a population of 350,000 and an area of 1,200 square miles split among 7 chiefdoms. The DHMT, GOAL, WHO, and CDC trained these new case investigators, and mentorship in the field reinforced the formal training, resulting in improved data collection quality. As new case investigators became operational, a rotation system was implemented to provide time off. This helped address surveillance efficiency, quality, and case investigator work fatigue.

Case investigators received training and mentorship to improve data collection quality.

Resolving compensation issues for case investigators was a protracted and complicated process. Initially, NERC paid all case investigators, with funding from the World Bank. GOAL advocated with NERC on behalf of case investigators, the visibility of which provided some comfort to case investigators. Ultimately, to alleviate ongoing challenges surrounding payments, GOAL secured DFID funding and NERC permission to pay all case investigators directly in both districts beginning in July 2015. Direct and timely payment considerably improved case investigator morale.

**Figure:**
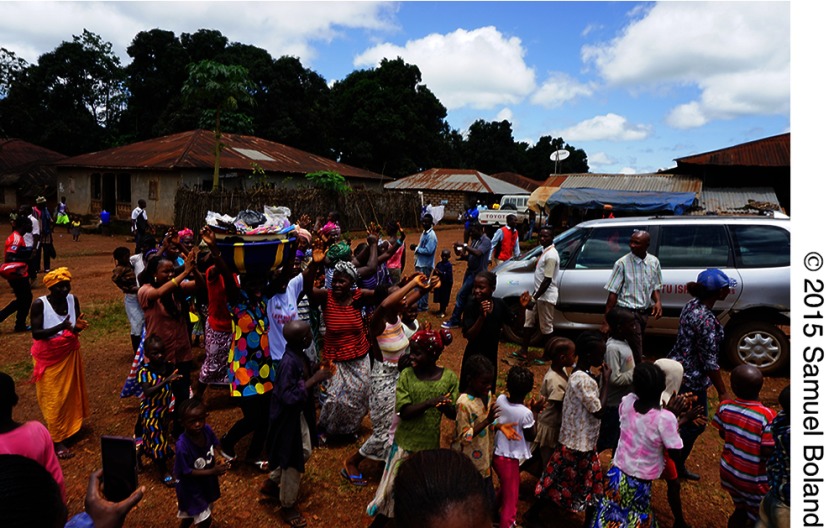
Community members in Kambia, Sierra Leone, welcome case investigators and their release from quarantine.

**FIGURE 3:**
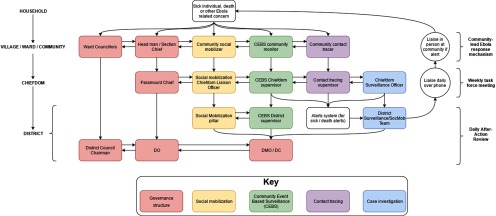
GOAL Ebola Virus Disease Response Structure, Sierra Leone Abbreviations: CEBS, community event-based surveillance; DC, District Coordinator; DMO, District Medical Officer; DO, District Officer. Note: Developed by Samuel Boland on behalf of GOAL.

Inter-pillar coordination meetings were established to create horizontal linkages between the pillars in Port Loko in January 2015, and in Kambia in April 2015. GOAL attempted to reinforce horizontal communication by developing an EVD response framework (Figure 3). Unfortunately, this structure was not fully realized in either district as it was developed late in the response. However, responses to future emergencies should consider similar coordination mechanisms that encourage both horizontal and vertical communication.

An EVD response framework was established to create linkages between pillars, but it was not fully realized as it was developed late in the response.

Leadership challenges in Kambia were mitigated to some degree through relationship building and regular coordination with technical leads and DERC management; however, case investigation management remained fragmented. To address the high proportion of EVD cases identified post-mortem or with no known source case, GOAL, WHO, and CDC developed an active surveillance strategy in Port Loko, which began in April 2015. This strategy targeted operational resources based on epidemiologic analysis of areas that were underreporting—an ideal convergence of operational and technical expertise. DERC leadership in Kambia decided not to employ this strategy as they considered it too difficult to operationalize. Although similar interventions occurred in the 2 districts, analysis of alert data from June 2015 to September 2015 (during Operation Northern Push, an effort by the NERC and DFID to eliminate EVD transmission from Port Loko and Kambia), showed statistically significant lower percentage increases in alerts in Kambia.^18^

## RECOMMENDATIONS

EVD case investigation in Port Loko and Kambia districts of Sierra Leone faced numerous environmental, infrastructural, sociocultural, political, and organizational obstacles and difficulties. GOAL's intervention, beginning in January 2015 in Port Loko and April 2015 in Kambia, addressed these issues to varying degrees of success in each district.

While the focus on technical support is indispensable in any outbreak, operational needs must be addressed and structures established to ensure the quality and systematic collection of surveillance data, as well as its storage, organization, retrievability, and communication. Without comprehensive and reliable data, effective technical support is limited. In future outbreaks, all of the following operational components must also be addressed within a politically and organizationally enabling environment:

- Ensure frontline staff receive timely compensation for and sufficient rest from their work, to boost staff morale, efficiency, and safety.
- Provide sufficient and context-appropriate transportation and communication resources, to ensure effective communication between field staff, their coordinators, and community members.
- Establish systematic data collection, storage, and retrieval systems, to ensure that any record can be effectively and efficiently accessed.
- Create formal linkages between frontline staff and the communities they work in to develop trust between them, thus increasing staff safety and investigation quality.
- Establish daily meetings between frontline staff and epidemiologists to ensure information and data quality and to provide opportunities for daily mentorship and training.
- Identify clear and appropriate leadership for operational chain of command.
- Give credence at all political and funding levels to operational foundations.

Technical support is indispensable in any outbreak but is limited unless operational needs are addressed and structures established to ensure the quality and systematic collection of surveillance data.

## Notes

**Peer Reviewed**

**First Published Online:** 2017 July 27
